# Supplementary material for: Suicide literacy and stigma among undergraduate students in Pokhara, Nepal: A cross-sectional study
Source: PLoS One. 2025 Sep 2;20(9):e0325617. doi: 10.1371/journal.pone.0325617 (PMC12404396; doi:10.1371/journal.pone.0325617)
Supplement: S1 Table — (DOCX) [file pone.0325617.s003.docx]

**Supplementary Analysis**

The regression analysis revealed significant associations between academic faculty and suicide stigma scores among undergraduate students. Compared to students from the Management faculty (reference group), those studying Engineering (B = -1.604, p = 0.020) and Humanities (B = -3.497, p = 0.003) reported significantly lower stigma, suggesting more accepting attitudes toward suicide.

**Supply table 1: Multiple Linear Regression Analysis of Factors Associated with Suicide Stigma (SOSS Stigma Average) Among Undergraduate Students in Pokhara, Nepal.**

| Variables | Unstandardized Coefficients | | Standardized Coefficients | t | Sig. | 95.0% Confidence Interval for B | |
| --- | --- | --- | --- | --- | --- | --- | --- |
|  | B | Std. Error | Beta |  |  | Lower Bound | Upper Bound |
| Faculty (Engineering) | -1.604 | 0.686 | -0.102 | -2.338 | 0.020 | -2.952 | -0.256 |
| Faculty (Law) | 1.411 | 1.249 | 0.047 | 1.130 | 0.259 | -1.042 | 3.863 |
| Faculty (Allied sciences) | 1.694 | 0.795 | 0.092 | 2.130 | 0.034 | 0.132 | 3.256 |
| Faculty (Education) | -0.476 | 1.272 | -0.015 | -0.374 | 0.709 | -2.974 | 2.022 |
| Faculty (Humanities) | -3.497 | 1.153 | -0.140 | -3.033 | 0.003 | -5.762 | -1.232 |
| Faculty (Science) | -0.494 | 0.841 | -0.025 | -0.587 | 0.557 | -2.146 | 1.159 |
| Age (Adult) | 0.708 | 0.944 | 0.034 | 0.750 | 0.453 | -1.145 | 2.562 |

The regression analysis identified that female students reported significantly lower scores (B = -1.033, p = 0.007), indicating lesser stigmatizing perceptions of suicide as an outcome of depression or isolation compared to males. Students from Engineering (B = -1.504, p = 0.006) and Humanities (B = -2.588, p = 0.002) faculties had significantly lower depression-related stigma, while those from Allied Sciences had higher stigma (B = 1.353, p = 0.017). Additionally, fourth-year students showed significantly higher depression stigma scores compared to second-year students (B = 1.134, p = 0.048). Other variables, including age group, accommodation status, and self-esteem, were not statistically significant. These results suggest that gender, faculty affiliation, and academic level are key factors influencing how students perceive suicide in terms of depression and isolation.

**Supp Table 2. Multiple Linear Regression Analysis of Factors Associated with Depression-Related Suicide Stigma (SOSS Depression Average) Among Undergraduate Students in Pokhara, Nepal.**

| Variables | Unstandardized Coefficients | | Standardized Coefficients | t | Sig. | 95.0% Confidence Interval for B | |
| --- | --- | --- | --- | --- | --- | --- | --- |
|  | B | Std. Error | Beta |  |  | Lower Bound | Upper Bound |
| Gender (female) | -1.033 | 0.382 | -0.116 | -2.701 | 0.007 | -1.784 | -0.282 |
| Age (Adolescent) | -0.439 | 0.668 | -0.030 | -0.657 | 0.512 | -1.752 | 0.874 |
| Accommodation (without family) | -0.690 | 0.400 | -0.069 | -1.726 | 0.085 | -1.475 | 0.095 |
| Faculty (Engineering) | -1.504 | 0.545 | -0.138 | -2.760 | 0.006 | -2.574 | -0.434 |
| Faculty (Law) | 0.691 | 0.922 | 0.033 | 0.749 | 0.454 | -1.121 | 2.502 |
| Faculty (Allied sciences) | 1.353 | 0.564 | 0.105 | 2.401 | 0.017 | 0.246 | 2.460 |
| Faculty (Education) | -0.777 | 0.915 | -0.036 | -0.848 | 0.397 | -2.574 | 1.021 |
| Faculty (Humanities) | -2.588 | 0.823 | -0.149 | -3.147 | 0.002 | -4.204 | -0.973 |
| Faculty (Science) | 1.089 | 0.652 | 0.080 | 1.670 | 0.095 | -0.192 | 2.371 |
| Education (3rd Year) | -0.475 | 0.538 | -0.051 | -0.883 | 0.378 | -1.532 | 0.582 |
| Education (4th Year) | 1.134 | 0.573 | 0.128 | 1.977 | 0.048 | 0.008 | 2.260 |
| Self-esteem (low) | 0.637 | 0.428 | 0.060 | 1.490 | 0.137 | -0.203 | 1.477 |
| Self-esteem (high) | -1.186 | 0.855 | -0.055 | -1.386 | 0.166 | -2.866 | 0.494 |

Female students showed significantly higher glorification scores than males (B = 1.022, p = 0.002), as did unmarried students compared to their married peers (B = 1.582, p = 0.013). Fourth-year students reported significantly greater glorification than second-year students (B = 1.448, p < 0.001). Additionally, students whose mothers were literate (B = 1.426, p = 0.017) were more likely to endorse glorifying attitudes, while students whose mothers were not employed had significantly lower glorification scores (B = -0.691, p = 0.028).

**Supp Table 3. Multiple Linear Regression Analysis of Factors Associated with Suicide Glorification (SOSS Glorification Average) Among Undergraduate Students in Pokhara, Nepal.**

| Variables | Unstandardized Coefficients | | Standardized Coefficients | t | Sig. | 95.0% Confidence Interval for B | |
| --- | --- | --- | --- | --- | --- | --- | --- |
|  | B | Std. Error | Beta |  |  | Lower Bound | Upper Bound |
|  |  |  |  |  |  |  |  |
| Gender(female) | 1.022 | 0.332 | 0.126 | 3.080 | 0.002 | 0.370 | 1.674 |
| Marital Status (unmarried) | 1.582 | 0.634 | 0.105 | 2.494 | 0.013 | 0.336 | 2.827 |
| Marital Status (Separated) | -1.236 | 2.018 | -0.026 | -0.612 | 0.541 | -5.199 | 2.728 |
| Education (3rd Year) | -0.686 | 0.403 | -0.080 | -1.701 | 0.090 | -1.477 | 0.106 |
| Education (4th Year) | 1.448 | 0.372 | 0.180 | 3.894 | 0.000 | 0.718 | 2.178 |
| Mother’s education (Literate) | 1.426 | 0.593 | 0.096 | 2.404 | 0.017 | 0.261 | 2.592 |
| Mother’s Employment (No) | -0.691 | 0.313 | -0.088 | -2.207 | 0.028 | -1.307 | -0.076 |

This regression analysis examined factors associated with suicide literacy (LOSS-SF total score) among undergraduate students. Compared to their counterparts, students living with family had significantly higher literacy scores (B = 0.434, p = 0.026). Faculty-wise, students from Engineering (B = -1.032, p < 0.001) and Education (B = -0.901, p = 0.042) reported significantly lower suicide literacy, whereas those from Law had significantly higher literacy (B = 1.039, p = 0.020) compared to students from the Management faculty (reference group). Fourth-year students had significantly higher literacy than second-year students (B = 0.552, p = 0.048). Moreover, students with low perceived family support had significantly higher literacy scores (B = 2.726, p = 0.026) compared to those with moderate support. Other variables, including gender, age, and most faculty or education levels, were not statistically significant.

**Supp Table 4. Multiple Linear Regression Analysis of Factors Associated with Suicide Literacy (LOSS-SF Total Score) Among Undergraduate Students in Pokhara, Nepal.**

| Variables | Unstandardized Coefficients | | Standardized Coefficients | t | Sig. | 95.0% Confidence Interval for B | |
| --- | --- | --- | --- | --- | --- | --- | --- |
|  | B | Std. Error | Beta |  |  | Lower Bound | Upper Bound |
| Gender(female) | -0.232 | 0.184 | -0.054 | -1.264 | 0.207 | -0.594 | 0.129 |
| Age (Adolescent) | -0.160 | 0.324 | -0.023 | -0.493 | 0.623 | -0.796 | 0.477 |
| Accommodation (family) | 0.434 | 0.194 | 0.090 | 2.238 | 0.026 | 0.053 | 0.816 |
| Faculty (Engineering) | -1.032 | 0.265 | -0.195 | -3.897 | 0.000 | -1.552 | -0.512 |
| Faculty (Law) | 1.039 | 0.447 | 0.102 | 2.324 | 0.020 | 0.161 | 1.916 |
| Faculty (Allied sciences) | 0.497 | 0.275 | 0.080 | 1.810 | 0.071 | -0.042 | 1.036 |
| Faculty (Education) | -0.901 | 0.443 | -0.087 | -2.035 | 0.042 | -1.770 | -0.032 |
| Faculty (Humanities) | -0.588 | 0.398 | -0.070 | -1.477 | 0.140 | -1.370 | 0.194 |
| Faculty (Science) | 0.040 | 0.317 | 0.006 | 0.127 | 0.899 | -0.582 | 0.663 |
| Education (3rd Year) | -0.186 | 0.260 | -0.041 | -0.715 | 0.475 | -0.698 | 0.325 |
| Education (4th Year) | 0.552 | 0.279 | 0.129 | 1.982 | 0.048 | 0.005 | 1.100 |
| MSPSS Family (low support) | 2.726 | 1.221 | 0.093 | 2.232 | 0.026 | 0.327 | 5.124 |
| MSPSS Family_(high support) | -0.412 | 0.404 | -0.043 | -1.020 | 0.308 | -1.205 | 0.381 |
