# Supplementary material for: Suicide literacy and stigma among undergraduate students in Pokhara, Nepal: A cross-sectional study
Source: PLoS One. 2025 Sep 2;20(9):e0325617. doi: 10.1371/journal.pone.0325617 (PMC12404396; doi:10.1371/journal.pone.0325617)
Supplement: Attachment — Submitted filename: Reviewer Response.docx [file pone.0325617.s004.docx]

Dear Editor,

We sincerely thank you and the reviewers for your valuable feedback and time dedicated to improving our manuscript. We have carefully considered each comment and have made revisions to address these suggestions. Below is a detailed response to each point raised:

Reviewer #1:

1. Two relevant previous studies are available that are missing from the manuscript:
a) Gupta AK, Sharma R, Sah RP, Sharma S, Jha A, Chapagai M, Saeed F, Shoib S. Cross‐cultural adaptation of Nepalese literacy and stigma of suicide scales (LOSS‐SF‐Nep and SOSS‐SF‐Nep) among Nepalese medical and nursing students. Brain and behavior. 2023 Dec;13(12):e3344.

b) Shah S, Neupane D, Sah KK. Literacy of suicide among doctors and nurses at a tertiary care hospital in Nepal. Journal of College of Medical Sciences-Nepal. 2022 Jun 30;18(2):93-102.

- Thank you for your valuable time and for suggesting the missing citation. We have included them in our revised discussion section, strengthening the discussion

2. LOSS-SF and SOSS-SF instruments seem to used in English language in your study, though they have been adapted in Nepali language before. [REF: Gupta AK, Sharma R, Sah RP, Sharma S, Jha A, Chapagai M, Saeed F, Shoib S. Cross‐cultural adaptation of Nepalese literacy and stigma of suicide scales (LOSS‐SF‐Nep and SOSS‐SF‐Nep) among Nepalese medical and nursing students. Brain and behavior. 2023 Dec;13(12):e3344.]

- Thank you for notifying us about the tool validation. The data for this study were collected during 2022, and at that time, the mentioned validation study was not published. Therefore, our research team consulted with some experts and ensured translation validity. The presence of a validated tool is crucial progress, and we have highlighted the existence of a validated tool in the discussion section. We, however, have not included this validation study in the method section.

3. The mean score of LOSS-SF-Nep was 6.36 ± 1.92 as per Gupta et al, 2021 which is higher than your findings. Your lower score could possibly be because using the instrument in Nepalese language could bring higher understanding of the questionnaire and accurate literacy.

- Thank you for your critical observation. We have included this difference in our discussion section and acknowledge the limitation of our study regarding the tools. We have further suggested that future researchers to use the mentioned validated Nepalese version of the tool in the discussion section.

4. Just like LOSS, SOSS does not seem to be validated or adapted for the study population. It is difficult to understand how the words like shallow. lost, disconnected, etc were communicated or understood by the study volunteers whose mother tongue is not English and more than one-third have studied below 8th standard! This technical error could have been easily avoided by using the validated scale.

- Thank you for your comment. As mentioned above, at the time of data collection, the validated tool was unavailable, and we had consulted with experts and had also pretested the tool before going into the field to address the issues of misinterpretation and translation validity. But we acknowledge the tool might had brings some reporting bias and have included it as a limitation of our study in light of your valuable feedback.

5. Your conclusion seem to be partially based on your findings. For example, you say that this study confirms that suicide literacy among Nepalese college students is low, and stigma remains a significant barrier to help-seeking behaviors. I have a question: Stigma is known barrier to help-seeking but how does this study conclude this statement? Similarly, when you say that Demographic variations, such as gender and academic year, further influence levels of stigma and literacy, it is prudent to ask if these variables influence or are associated?

- Thank you for the suggestion. We have received the conclusion, making it more inline with our findings.

Reviewer #2:

What all undergrad courses were included? any exclusion? since under graduate medical student might be more aware of suicide as already included in course curriculum? does it affect study result?

- Thank you for your query. We have covered all six faculties available insight Pokhara Metropolitan for better representation. Considering the word economy we had not mentioned each of the processes, we thank you for helping us clarify this aspect. We had representation from Engineering (including BSc programs in Civil, Electrical, Mechanical, and Computer Engineering programs), Management (including BBA, BBS, and BBA-BI), and Law (BALLB and LLB). Allied Sciences refers to health-related disciplines such as Medicine, Dental, Nursing, Public Health, Pharmacy, physiotherapy, optometry, and Medical Laboratory Technology, optometry. Sciences refers to pure science disciplines, including BSc programs in Chemistry, Physics, Biology, Microbiology, and Forestry. Humanities were also represented through programs such as BDevS, Arts, B.Ed, and Humanities courses.
- In this study, the students enrolled in the first year were excluded, as they had just joined their courses and were not regular attendees. There were no exclusions made based on academic course.
- In regard to the presence of suicide related courses in undergraduate medical students, certainly it can be assumed that undergraduate students enrolled in medicine, public health, nursing, Pharmacy, etc, have certain sessions related to suicide, and this might be associated with their literacy and/or stigma related to suicide. This is one of the reasons we did not make any exclusions in regards to academic courses, as it allowed us to check if there is any statistically significant difference in stigma and literacy related to suicide across academic disciplines. We have presented this finding in our Table 5 and have further revised the discussion section.

2. Study mentions nature of college. any significance of nature of college?

- Thank you for your query. The nature of the college is usually private or public. However, there was no significant difference in stigma and literacy related to suicide across the nature of the college.

3. consent verbal/written of parents not clearly mentioned?

- Thank you for notifying this missing information in our sample. only one student was 17 years old, for whom we had acquired parental consent by providing a consent form to the student a few days before data collection. For all students, written informed consent was secured day of data collection before the start of data collection.

4. Grammatical errors , typological errors found.
