# Supplementary material for: Suicide literacy and stigma among undergraduate students in Pokhara, Nepal: A cross-sectional study
Source: PLoS One. 2025 Sep 2;20(9):e0325617. doi: 10.1371/journal.pone.0325617 (PMC12404396; doi:10.1371/journal.pone.0325617)
Supplement: Attachment — Submitted filename: Response to Reviewers.docx [file pone.0325617.s005.docx]

Dear Editor,

Dear Editor,

We sincerely thank you and the reviewers for your valuable feedback and the time dedicated to improving our manuscript. We have carefully considered each comment and made the necessary revisions to address the suggestions.

As suggested by the editors, we have explicitly stated that:
“The funders had no role in study design, data collection and analysis, decision to publish, or preparation of the manuscript.”

Below is a detailed response to each point raised by the reviewers.

1. Page 5: The statement ‘to account for potential variability, the sample size was adjusted by applying a design effect of 1.5 times the sample size, resulting in 576 required samples’ requires revision. e.g. ‘A design effect of 1.5 was applied to the sample size to account for clustering in the sampling design.

- Response: Thank you for the suggestion. We have revised this section accordingly.

2. Page 5: The questionnaires' language version and the validity and reliability information of their translated versions are to be provided. The scoring method for the questionnaires to be provided.

- Response : Thank you for the comment. The original versions of LOSS, SOSS, and SIDAS were used in the study and we have cited them within the manuscript while mentioning them. At the time this study was implemented, there were no validated Nepalese versions of these tools; thus, we took the help of bilingual mental health experts to translate these tools in Nepali language and also conducted a pretest to ensure translation validity. We have mentioned this in our manuscript. Thank you for your comment, we have added the use Nepali version of the tool in the manuscript.

3. Page 5: A statement/ description on missing values and the method of handling the missing data (if any) is to be mentioned.

- In this study, we had approached 600 students, and 597 students provided a complete response. This was above the minimum required sample size so the data from 597 students were included in this study. We have added this revision in our manuscript.

4. Page 6: The citation of the statistical software used and the publisher is to be provided.

- Response: Thank you for your suggestion. We have cited the statistical software used

5. A statement on the fulfillment of assumptions for the statistical tests used is to be stated.

- Response: Thank you for your suggestion, we have revised the analysis section to clearly state the statistical test used in the study.

6. Page 6: The purpose of using the specific statistical test is to be clearly mentioned. The outcome variables are to be stated in the statistical analysis section.

- Response: The normality of the data was evaluated using the Shapiro-Wilk normality test, a common statistical tool used to assess the normality assumption of a dataset. As the data followed normal distribution independent student t-tests and ANOVA were performed. We have revised the analysis section to clarify this.

7. Page 6: One or two-tailed test is to be mentioned. The specific name/type of ANOVA is to be stated. For ANOVA, post hoc analyses are to be presented/highlighted.

- Response: Statistical significance was set for a two-sided P-value < 0.05. One-way analysis of variance (ANOVA), and post-hoc analysis was performed using the Turkey post-hoc test with Games-Howell.

8. Table 1: The overall sample size (N) is to be clearly stated. The variable 'academic year' includes only 595 students instead of 597. If there is missing data, this should be acknowledged and explained.

- Response: Thank you for notifying the typo error in data distribution of academic year, we have corrected it in the revised manuscript.

9. Table 4: The word agreement is to be denoted e.g. what agreement refers to.

- Response: The word agreement denotes participants' response in SOSS, where they responded agreeing or strongly agreeing with the respective statement.

10. Table 6 title requires revision to be more specific (as compared to Table 5 title).

- Response: Thank you for your suggestion. We have revised it accordingly.

11. Page 6: It was mentioned ‘95% confidence interval’, but there was no 95% CI presented in the results section.

- Response: Thank you for your comment. We have removed the statement regarding 95% confidence interval from the methods section.

12. Tables 5 and 6: Statistical tests are to be denoted in the table footnote.

- Response: Thank you for the suggestion. We have denoted the statistical tests in the table footnote.

13. All statistical tests used in the results are to be mentioned in the statistical analyses in the method section.

- Response: We have mentioned all the statistical test used in the method section.

14. The name of the correlation test is to be stated.

- Response: Thank you for your suggestion. The name of the correlation test has been mention in the manuscript

15. Page 11: Table 5 is to be cited in the text.

- Response: Thank you for notifying this missing citation. We have cited a table in the text.

16. Page 12: The sentence ‘The comparative analysis revealed that individuals’ requires revision.

- Response: Thank you for your suggestion. We have revised it accordingly.

17. Page 13: The paragraph section on correlation requires revision by adapting the words weak and moderate correlation into it where applicable.

- Response: Thank you for your valuable suggestion. We have revised the paragraph section on correlation by incorporating the terms weak, moderate, and high correlation where applicable.

18. Page 13: Related to peer support (r = 0.392) is an incorrect statement. The sentence requires improvement.

- Response: Thank you for the suggestion. We have revised the statements.

19. Page 16: Correlation value is to be provided where appropriate.

- Response : Thank you for your suggestion. We have included the correlation values where appropriate

20. Further statistical analyses e.g. regression, could be performed.

- Response: Thank you for your valuable suggestion regarding the inclusion of regression analysis. We acknowledge that regression analysis could provide insights into predictive relationships. However, our primary objective was to explore and compare suicide literacy and stigma levels across different socio-demographic and psychosocial groups. The study was exploratory in nature, and we used appropriate comparative and correlational analyses (e.g., t-tests, ANOVA, and Pearson correlation) to meet this objective. We respectfully chose not to perform regression analysis in this manuscript and we thank you for your valuable suggestion and kind consideration.

21. The word p-value in the tables could be replaced with p

- Response: Thank you for your suggestion. We have revised accordingly.
