# Supplementary material for: Suicide literacy and stigma among undergraduate students in Pokhara, Nepal: A cross-sectional study
Source: PLoS One. 2025 Sep 2;20(9):e0325617. doi: 10.1371/journal.pone.0325617 (PMC12404396; doi:10.1371/journal.pone.0325617)
Supplement: Attachment — Submitted filename: Response letter.docx [file pone.0325617.s006.docx]

Dear Editor,

We sincerely thank you and the reviewers for your valuable feedback and the time dedicated to improving our manuscript. We have carefully considered each comment and made the necessary revisions to address the suggestions.

As suggested by the editors, we have explicitly stated that:
“The funders had no role in study design, data collection and analysis, decision to publish, or preparation of the manuscript.”

Below is a detailed response to point raised by the reviewers.

The authors’ justification for not performing regression analysis, stating that "our primary objective was to explore and compare suicide literacy and stigma levels across different socio-demographic and psychosocial groups " does not align well with the stated objective of the study: "This study examines suicide literacy and stigma among undergraduate students and its associated factors.' This discrepancy should be addressed, and the objective should be revised for clarity and consistency. The discussion needs to be revisited due to the exploratory nature of the study, as stated by the authors.

Response: Thank you for your insightful comment. We have performed multiple linear regression analyses to identify factors associated with suicide stigma and literacy. The results of these analyses, including full coefficient tables for all models (SOSS-Stigma, SOSS-Depression, SOSS-Glorification, and LOSS-SF), are provided in Supplementary File S1.
